# Puerperal Convulsions—Treatment

**Published:** 1874-05

**Authors:** 


*THE MEDICAL RECORD—December*, 1873.

TREATMENT OF PUERPERAL CONVULSIONS.—The plan of treatment adopted by Prof. T. Gaillard Thomas, of New York, is essentially embraced in the following: When a toxæmic condition of the blood is recognized in the pregnant woman from the presence of albumen in the urine, keep the mucous tract of alimentary canal very active, prevent the woman from eating a large amount of animal food, and guard the skin against atmospherical changes in tempearature.

In the worst forms of uræmic toxæmia premature delivery will be required, and it should be accomplished at the end of the eighth month, unless the life of the mother is seriously jeopardized previous to this time. The child will almost surely die if left to the end of the nine months, and the life of the mother is greatly risked by the same postponement.

Venesection and chloroform are the two means for stopping this kind of convulsions. Now bring on labor, and remove the child as soon as you can do so safely; but you are not obliged to deliver immediately.

If the pains are of moderate severity, the case may be left to nature chiefly until dilation has taken place sufficient to admit of the use of the forceps or a resort to version, and either of these operations will be performed according to the circumstances of the case. There is generally no hurry about the delivering, for the woman is under the influence of chloroform.

The essential features of the plan of treatment adopted by Prof. Edmund R. Peaslee, of New York, are as follows: If, upon examination of the urine, albumen is found present in quantities sufficient to give any apprehensions with regard to its toxæmic effect upon the blood, the first indication is to make the kidneys act. This may be done by the application of dry cups over the region of the kidneys; sometimes a plaster of some kind is sufficient; and very generally acetate of potash will produce sufficient diuresis if other measures fail. If there is an œdematous condition of the extremities, a pretty sharp drastic purgative may be given, and repeated consistent with the effect and general strength of the patient.

But we will suppose that the woman suddenly, in the progress of her pregnancy, has a convulsion. The first thing to be done is to place the woman under the influence of chloroform, for the purpose of preventing the further occurrence of convulsions. She may be held under its influence for almost any length of time if it is nicely and carefully administered, and not carried to the extent of producing stertorous breathing.

If the veins about the face are full and turgid, it may be well to take a pint of blood by venesection, but I have not found it necessary to resort to this measure during the last fifteen years. It is done with the view of saving the brain—relieving the pressure upon the great nervous centers.

If the chloroform prevents the convulsions, sufficient delay may be made to permit the head to come down, so that it can be readily delivered with the forceps; but if, after dilatation, there is necessity for immediate delivery, or there is any doubt with regard to the efficiency of the labor-pains while the hand is in the uterus, deliver by version.

The first principle in the management of a case of puerperal convulsions is to look for the safety of the mother. Get rid of the child as soon as possible, but at the same time do not risk the life of the mother.

Prof. A. L. Loomis, M.D., of New York.—The plan of treatment which was particularly recommended in the treatment of cases of acute uræmia, was to produce diuresis by the use of an infusion of digitalis; and if convulsions appeared, to control them by the use of morphine administered hypodermically.

Dr. Anderson, ex-President of the Academy, remarked that he believed "vent must be given somewhere." In one case which he treated by the use of ether inhalations, with only partial success in controlling the convulsions, he gave the patient grs. xx of calomel, and followed it soon after with an injection of turpentine and soapsuds, and the result was an overflow of the bed, and a complete subsidence of the convulsion.

Dr. Baxter remarked that he was heartily sick of the use of chloroform, and that he relied upon infusion of digitalis in ℥ss. doses every two or three hours, and blood-letting.

Prof. White, of Buffalo.—I am in favor of the use of opium, but I have never felt that it was important to give it hypodermically.

I believe that opium is beneficial from the fact that it relieves cerebral congestion.

Again, I think it is important to unload.

The cathartic which I prefer of all others is the croton oil. It is easily administered, is efficient, and safe if properly administered.

Another benefit which I think arises from the use of croton oil as a chathartic, is, that it irritates the mucous membrane of the entire intestinal canal, and in this way operates as a revulsive. This I regard as an important benefit. I still think that there are cases of convulsions where the abstraction of blood is important. I would therefore still resort to blood-letting occasionally, and the indications are plain which would demand the employment of this measure. The larger proportion of women, however, I believe will not require it.

